# Changes in *Biceps femoris* Transcriptome along Growth in Iberian Pigs Fed Different Energy Sources and Comparative Analysis with Duroc Breed

**DOI:** 10.3390/ani11123505

**Published:** 2021-12-08

**Authors:** Rita Benítez, Yolanda Núñez, Miriam Ayuso, Beatriz Isabel, Miguel A. Fernández-Barroso, Eduardo De Mercado, Emilio Gómez-Izquierdo, Juan M. García-Casco, Clemente López-Bote, Cristina Óvilo

**Affiliations:** 1 Departamento de Mejora Genética Animal, Instituto Nacional de Investigación y Tecnología Agraria y Alimentaria (INIA-CSIC), 28040 Madrid, Spain; rmbenitez@inia.es (R.B.); nunez.yolanda@inia.es (Y.N.); fernandez.miguel.inia@gmail.com (M.A.F.-B.); garcia.juan@inia.es (J.M.G.-C.); 2 Department of Veterinary Sciences, Faculty of Biomedical, Pharmaceutical and Veterinary Sciences, University of Antwerp, B-2610 Wilrijk, Belgium; mayher@live.com; 3 Departamento de Producción Animal, Facultad de Veterinaria, Universidad Complutense de Madrid, 28040 Madrid, Spain; bisabelr@pdi.ucm.es (B.I.); clemente@vet.ucm.es (C.L.-B.); 4 Centro de Pruebas de Porcino ITACYL, Hontalbilla, 40353 Segovia, Spain; ita-merpened@itacyl.es (E.D.M.); gomizqem@itacyl.es (E.G.-I.)

**Keywords:** nutrigenomics, diet, breed, age, interaction, muscle tissue, Iberian pig, Duroc pig, transcriptome, growth and development

## Abstract

**Simple Summary:**

The genetic mechanisms that regulate biological processes, such as skeletal muscle development and growth, or intramuscular fat deposition, have attracted great interest, given their impact on production traits and meat quality. In this sense, a comparison of the transcriptome of skeletal muscle between phenotypically different pig breeds, or along growth, could be useful to improve the understanding of the molecular processes underlying the differences in muscle metabolism and phenotypic traits, potentially driving the identification of causal genes, regulators and metabolic pathways involved in their variability.

**Abstract:**

This experiment was conducted to investigate the effects of developmental stage, breed, and diet energy source on the genome-wide expression, meat quality traits, and tissue composition of *biceps femoris* muscle in growing pure Iberian and Duroc pigs. The study comprised 59 Iberian (IB) and 19 Duroc (DU) animals, who started the treatment at an average live weight (LW) of 19.9 kg. The animals were kept under identical management conditions and fed two diets with different energy sources (6% high oleic sunflower oil or carbohydrates). Twenty-nine IB animals were slaughtered after seven days of treatment at an average LW of 24.1 kg, and 30 IB animals plus all the DU animals were slaughtered after 47 days at an average LW of 50.7 kg. The main factors affecting the muscle transcriptome were age, with 1832 differentially expressed genes (DEGs), and breed (1055 DEGs), while the effect of diet on the transcriptome was very small. The results indicated transcriptome changes along time in Iberian animals, being especially related to growth and tissue development, extracellular matrix (ECM) composition, and cytoskeleton organization, with DEGs affecting relevant functions and biological pathways, such as myogenesis. The breed also affected functions related to muscle development and cytoskeleton organization, as well as functions related to solute transport and lipid and carbohydrate metabolism. Taking into account the results of the two main comparisons (age and breed effects), we can postulate that the Iberian breed is more precocious than the Duroc breed, regarding myogenesis and muscle development, in the studied growing stage.

## 1. Introduction

Pig meat is widely consumed worldwide and comprises one of the main sources of protein for human nutrition. Modern pig production is mostly established on highly selected genotypes, which are produced in intensive systems, focused on the efficient acquisition of meat. Nevertheless, in the south of Europe, this production system exists alongside autochthonous, less efficient breeds, as is the case of the Iberian pig breed. The traditional-based production systems are attaining important technical and commercial interest because of their use in the production of high-quality meat and derived products [1], and they are characterized by limited efficiency and muscle accretion, together with high fat accumulation (including intramuscular fat) and adaptation to harsh environmental conditions [2]. The Iberian pig traditional production system is a good example of a sustainable system [3], based on the *ad libitum* intake of natural resources in a characteristic extensive system (“montanera”). Moreover, this breed is characterized by high residual feed intake and a peculiar fatty acid (FA) profile [1], with a high concentration of monounsaturated fatty acids (MUFA), which leads to distinctive organoleptic and technological characteristics of its meat and markedly influences the excellent quality of its dry-cured products. These characteristics are due to its genetic predisposition [4,5] and the traditional production system.

Nowadays, the Iberian pig may also be reared under intensive conditions, based on local agriculture, and with a high standard of quality characteristics and welfare conditions. In this case, both purebred Iberian and Duroc pigs may be used as terminal sires, with the aim of improving reproductive and growth performances, and primal cut yields [4,6]. It has been reported that the use of Duroc genetics may negatively affect some quality characteristics, such as intramuscular fat (IMF) and oleic acid (OL), and other MUFA concentrations [7,8,9]. The differences in muscle growth and composition between DU and IB pigs are a potential model for the study of the mechanisms underlying muscle phenotype differences, regarding tissue development and fat accretion. Additionally, in intensive production systems, alternative oleic acid-enriched diets are being essayed in order to mimic the traditional feeding in “montanera”, potentially influencing tissue metabolism through nutrigenomic effects.

IMF and FA compositions are highly dependent on breed and diet [10,11]. At the cellular level, the number of adipocytes (hyperplasia) and their size (hypertrophy) within muscle fibers also determine IMF content [12]. Preadipocyte differentiation starts in the prenatal period and persists during early postnatal development, but this process decreases along growth [13], and in the later growth and adult period, adipocyte hypertrophy is the preponderant process affecting IMF content [14]. Moreover, in the early periods, the skeletal muscle mass also grows through two analogous processes, hyperplasia and hypertrophy, which vary according to the physiological conditions [15]. Thus, the growth period is essential in the investigation of adipocyte differentiation and the skeletal muscle development processes. In fact, age is the main factor affecting gene expression [4,16,17]. In addition, in nutrigenomic studies, the timing of sampling after the start of the dietary treatment may have a deep influence on the differential expression results [18].

Both developmental timing and metabolic and physicochemical features differ between muscles [19]. The *biceps femoris* (BF) muscle is the main lean mass within the ham and is characterized by high oxidative ability, due to the relatively higher content of type I muscle fibers than other muscles, such as the *Longissimus dorsi* (LD) muscle [20,21], also showing important differences regarding IMF content.

Several transcriptomic studies have compared lean *versus* fatty pig breeds in different developmental stages. Some studies compared different Chinese fatty pig breeds to lean western ones, such as the comparison between Tongcheng and Yorkshire pigs across 11 developmental stages [16], between Lantang and Landrace pigs during muscle development [15], or between Wei and Yorkshire pigs [22]. Additionally, some previous studies, based on microarray [23] or RNA-Seq technology [4,24], have pointed out transcriptomic differences when comparing pure Iberian pigs to Duroc crossbred Iberian pigs. In recent work [5], we studied the effects of breed, diet, and their interactions with the adipose tissue transcriptome in pure Iberian and Duroc growing pigs, bred in identical conditions. The results of this work indicated a strong effect of breed and a moderate effect of diet on adipose tissue gene expression. This effect was different in both breeds, and affected relevant biological functions and pathways related to growth and tissue development, inflammatory response, immune cell trafficking, and carbohydrate and lipid metabolism.

The present work employed the same experimental animals [5] and evaluated the transcriptional regulation of the BF muscle at two developmental stages in growing Iberian pigs. We evaluated the effects of age (within the Iberian breed), breed (IB vs. DU at the growing stage), and diet in both breeds (6% oleic sunflower oil or carbohydrates as the energy source) on the BF muscle transcriptome.

## 2. Materials and Methods

### 2.1. Ethics Statement

All experiments were carried out in accordance with the Spanish Governance for Protection of Animals used in Research, RD 53/2013, which covers the European Union Directive 2010/63/EU about the protection of animals used in experimentation. The project was approved on 20 March 2015, by the *Comunidad de Madrid* animal welfare and protection committee, with reference number PROEX-007/15.

### 2.2. Animals and Sampling

The present study was carried out at the facilities of the Pig Test Center ITACYL (Hontalbilla, Segovia, Spain) and comprised a total of 78 animals, including 59 Iberian (Torbiscal strain) and 19 Duroc males born in 32 contemporary litters (26 Iberian litters and 6 Duroc litters), who started the experiment at 19.9 kg (standard deviation (SD) = 3.8 kg) (LW). Iberian litters had 2 to 4 male piglets and Duroc litters had 3 to 5 male piglets. At 10 weeks of age (SD = 1.6 days), the animals were allocated to two experimental groups, by distributing the animals coming from the same litter into the two groups, and fed two different isocaloric and isoproteic diets (3.3 Kcal of digestible energy and 15.6% of crude protein). Both diets differed in the energy source; the experimental diet was enriched with 6% high oleic sunflower oil (HO) and the standard diet contained carbohydrates as the energy source (CH). Feed and fresh water were provided ad libitum. Pigs were kept under identical management conditions, housed in 20 pens within the same experimental shed (3–5 pigs/pen; 1 m^2^ pig^−1^), with a concrete floor and straw bedding. Iberian animals were distributed in 16 pens (8 for CH diet and 8 for HO diet) and Duroc animals were distributed in 4 pens (2 for CH diet and 2 for HO diet ). Temperature was recorded with a mean of 23.8 °C throughout the experiment. Twenty-nine Iberian pigs (HO diet *n* = 14 and CH diet *n* = 15) were slaughtered after seven days of treatment (transition piglets), at an LW of 24.1 kg (SD = 3.3 kg). Thirty Iberian pigs (HO diet *n* = 17 and CH diet *n* = 13) and all nineteen Duroc pigs (HO diet *n* = 10 and CH diet *n* = 9) were slaughtered after forty-seven days of treatment (grower pigs), at an LW of 50.2 kg (SD = 7.5 kg) and 51.2 kg (SD = 5.09 kg) for Iberian and Duroc pigs, respectively. Feed composition is shown in Table 1.

The animals were sampled immediately after euthanasia performed by stunning and exsanguination in compliance with RD 53/2013 standard procedures.

### 2.3. Muscle Tissue Composition, Meat Tenderness and Histological Analyses

*Biceps femoris* samples from all 78 animals (29 Iberian transitional piglets, 30 Iberian growers and 19 Duroc growers) were taken after slaughter. A slice of the muscle was taken from its central part (60 ± 2 mm) and divided into two slices (30 ± 5 mm); one slice was for the composition analysis, another for textural analysis and a small portion of 100 mg for RNA extraction. The slices were cut perpendicular to the longitudinal axis of the muscle. The IMF content of BF muscle was quantified using the method proposed by Segura et al., 2014 [25], based on gravimetrical determination of lipid content. Lipid extracts from BF muscle were separated into neutral lipids (NL), polar lipids (PL) and free lipids (FL) using aminopropyl minicolumns, following the aforementioned method. Fat extracts were methylated in the presence of sulfuric acid and analyzed by gas chromatography as described elsewhere [26] using a Hewlett Packard HP-6890 gas chromatograph (Avondale, PA, USA) equipped with a flame ionization detector and capillary column (HP-Innowax, 30 m 0.32 mm i.d. and 0.25 μm polyethylene glycol—film thickness). A temperature program of 170 to 245 °C was used. The injector and detector were maintained at 250 °C. The carrier gas (helium) flow rate was 2 mL/min. For the identification of each fatty acid, standard patterns were used (Sigma, Alcobendas, Madrid, Spain). Results were expressed as grams per 100 g of detected FAMEs (fatty acid methyl esters).

Regarding meat quality analysis, a portion of each fresh muscle from 49 grower pigs (30 Iberian and 19 Duroc) was divided into several parts for the subsequent determinations. The samples were vacuum packed in nylon/polyethylene bags and stored at −20 °C for thawing and cooking loss, instrumental color (lightness L*, redness a* and yellowness b*) and cooked meat shear force determinations. The meat quality analyses were carried out following the procedures described in [27].

Regarding the histological analysis, a portion of each muscle tissue from 49 growers (30 Iberian and 19 Duroc) was divided into 2 to 4 cm^2^ sections. The sections were fixed in 10% neutral buffered formalin. Then the specimens were embedded in paraffin, cut at 4 μm and stained with hematoxylin and eosin for routine examination [28]. The measurement of total adipose tissue surface in relation to the skeletal muscle surface was performed, as well as the measurement of the diameter of a total of 30 adipocytes per sample, discarding those with a smaller diameter (to avoid measuring adipocytes that were cut at the margins of the cell). These measurements were made with the Image J™ software (U.S. National Institutes of Health, Bethesda, MD, USA), on photomicrographs taken with a Leica ICC50W™ camera coupled to a Leica DM1000™ microscope (Leica, Mannheim, Germany).

### 2.4. Statistical Analyses of Phenotypic Data

The influence of breed, age and diet on FA composition was separately analyzed for each FA or FA index, within each lipid fraction. The analysis of the different effects on the FA composition, meat quality determinations, IMF content and size of adipocytes of BF muscle was performed with a linear model fitting as fixed-effects age, diet and the age–diet interaction (for Iberian transition and grower animals’ data, refer to model 1), and breed, diet and the diet–breed interaction (for Iberian and Duroc grower animals’ data, refer to model 2), and litter, pen and residual as random effects (in both models). Pen was nested to (Diet × Age) and (Diet × Breed) in the respective models. The animal was the experimental unit for all analyses. FA composition analyses were carried out using JMP^®^ Pro 13 (SAS Institute Inc., Addison, TX, USA) and the rest of the analyses were carried out using the MIXED procedure of SAS 9.4 (SAS Institute Inc., Cary, NC, USA). The results were considered to be significant at *p*-value < 0.05 and data were log transformed when necessary to meet normality and homoscedasticity criteria.

Model 1 is as follows:

(1) $$y_{ijklm}=Diet_{i}+Age_{j}+{\left(Diet\;\times \;Age\right)}_{ij}+Litter_{k}+Pen{\left(Diet\;\times \;Age\right)}_{l\left(ij\right)}+e_{ijklm}$$

Model 2:

(2) $$y_{ijklm}=Diet_{i}+Breed_{j}+{\left(Diet\;\times \;Breed\right)}_{ij}+Litter_{k}+Pen{\left(Diet\;\times \;Breed\right)}_{l\left(ij\right)}+e_{ijklm}$$

### 2.5. RNA Isolation, Library Construction and Sequencing

Muscle samples were maintained at −80 °C until gene expression analysis. For the transcriptome study, 36 muscle samples were used, corresponding to 12 Iberian animals belonging to the first slaughter (6 animals of each diet group) and 24 animals belonging to the second slaughter (12 animals of each breed, with 6 animals corresponding to each dietary group). The animals were randomly selected to perform transcriptomic analysis, representing all available litters. Total RNA was isolated from 50–100 mg samples of BF using the RiboPureTM RNA isolation kit (Ambion, Austin, TX, USA), following the manufacturer’s recommendations. The obtained RNA was quantified using NanoDrop equipment (NanoDrop Technologies, Wilmington, DE, USA), and the RNA quality was assessed with an Agilent 2100 bioanalyzer device (Agilent Technologies, Palo Alto, CA, USA) and submitted to the CNAG_CRG (Centro Nacional de Análisis Genómico, Barcelona, Spain). Libraries were prepared using the TruSeq mRNA-Seq sample preparation kit (Illumina Inc., Cat. # RS-100-0801, San Diego, CA, USA) according to manufacturer’s protocol. Each library was paired-end sequenced (2 × 75 bp) using TruSeq SBS Kit v3-HS, on a HiSeq2000 platform (Illumina, San Diego, CA, USA).

### 2.6. Bioinformatic Analyses

FastQC software (version 0.11.8) (Babraham Institute, Cambridge, UK; www.bioinformatics.babraham.ac.uk/projects/fastqc/; accessed on 13 March 2021) was employed to assess the quality of raw sequencing data. TrimGalore (version 0.5.0) (Babraham Institute, Cambridge, UK; https://www.bioinformatics.babraham.ac.uk/projects/trim_galore/; accessed on 13 March 2021) was used to qualitatively trim data with default settings, and to remove the Illumina sequencing adapters and poly A and poly T tails (stringency of 6 bp, -s 6) keeping only paired-end reads where both pairs were longer than 40 bp.

Filtered reads were mapped against the pig reference genome (Sscrofa11.1) using TopHat (v.2.1.0) [29] with Bowtie2 (v.2.2.7.0) applying default settings, except those reads that were first aligned to the ENSEMBL (11.1.90) [30] transcriptome annotation (-G option). Additionally, the distance between both pairs was set to 100 bp (inner-mean distance) and the standard deviation to 150 bp.

In order to confirm that the mapping had been carried out correctly, quality control was performed in all samples using two tools, Samstats [31] and Qualimap [32]. These programs provide information on the quality score of each mapped read, its length, and depth of mapping, composition and quality of the bases.

Resulting BAM files were analyzed with HTSeq-count (version 0.11.1) [33] to count and merge reads based on overlapping paired-end reads, resulting in Gcount files.

### 2.7. Differential Expression Analysis

The differential expression analyses were carried out in the R environment (R Core team) with the “DESeq2”package [34] that offers a complex method for gene-level analysis of RNA-seq data and uses Benjamini–Hochberg (BH) adjustment. DESeq2 was used at BH-adjusted *p*-value ≤ 0.05 for the breed effect, BH-adjusted *p*-value ≤ 0.1 for the diet effect and BH-adjusted *p*-value ≤ 0.01 for the age effect and FC ≥ 1.5 in all of them. This software supports complex experimental designs in addition to simple two-group setups. With DESeq2 software, RNA-seq read counts were modeled by generalized linear models including the breed, age and diet effects. The age and breed effects were tested including the diet effect in the model. The diet effect was evaluated within each breed and at each age.

### 2.8. Validation by qPCR

RNA obtained from the 36 samples used in the RNA-Seq assay was used to perform the technical validation of the differential expression of 12 genes that were affected either by the breed or by the age. This technical validation was performed by studying the Pearson correlation between the expression values obtained from RNA-seq data (counts) and the normalized gene expression data obtained by RT qPCR. To validate the global RNA-Seq results, the concordance correlation coefficient (CCC) [35] was calculated between the FC values estimated from RNA-Seq and qPCR expression measures for the 12 genes analyzed by the two technologies. The method proposed by Steibel et al., 2009 [36], was employed for the statistical analysis of qPCR gene expression data, following the procedure explained in other studies [5,9]. The *p*-values < 0.05 were considered statistically significant.

The expression of the genes *FASN*, *ME1*, *NR4A3*, *PON3*, *LEP*, *MSTN*, *IGF2*, *PVALB*, *DAPK3*, *GPX2*, *MTUS2* and *MGLL* was quantified. Primer pairs were designed using Primer Select software (DNASTAR, Madison, WI, USA) from the available GENBANK and/or ENSEMBL sequences. Primer pairs covered different exons to assure the amplification of the cDNA. Information on primer sequences and efficiency are indicated in Table S1. The most stable endogenous genes out of *GAPDH*, *ACTB*, *TBP*, *PPIA*, and *B2M* were selected for data normalization. The stability of the endogenous genes was tested with the Genorm and Normfinder softwares [37,38]. The *ACTB* and *PPIA* genes were selected as the most stable endogenous genes. Normalization of gene expression data was performed with Genorm software.

### 2.9. Functional Interpretation

Ingenuity Pathway Analysis software (IPA, QIAGEN Redwood City, CA, USA) was used to identify and characterize biological functions, canonical pathways and regulatory and causal networks affected by the DEGs [39]. IPA software was employed following the procedures described in Benítez et al., 2019 [5].

## 3. Results and Discussion

### 3.1. Age, Breed and Diet Effects on Phenotype

The effects of breed and diet on carcass traits and adipose tissue composition have been previously described [5,12]. Briefly, the main phenotypic traits related to fatness and premium cut yields were strongly different between breeds, as expected. Higher backfat thickness and average feed intake were observed in the Iberian pigs, whereas higher ham weight was registered in the Duroc pigs. On the other hand, it is noteworthy that, although the Iberian animals showed higher fatness and appetite, no significant difference was observed in body weight, suggesting higher muscle development in Duroc pigs [4,5,8]. The two dietary groups (HO and CH) showed similar body weight, backfat thickness, and ham weight at the end of the experiment. FA composition was studied at the following two different locations: subcutaneous backfat and ham fat. Regarding backfat FA composition, the main breed effects were a higher saturated fatty acid (SFA) content in the Iberian pigs, and higher MUFA and polyunsaturated fatty acids (PUFA) in the Duroc pigs. No difference between breeds was observed in oleic acid concentration. Correspondingly, the main breed effects in regards to ham fat were a higher SFA content in the Iberian pigs and higher PUFA in the Duroc pigs. In both locations (backfat and ham fat), a marked effect of dietary intervention was detected for SFA and MUFA concentrations. The HO feed exhibited a higher MUFA content, while a higher SFA content was observed in pigs fed the CH feed. No significant effect of litter or pen was observed on any of the studied traits.

**Figure 1:**
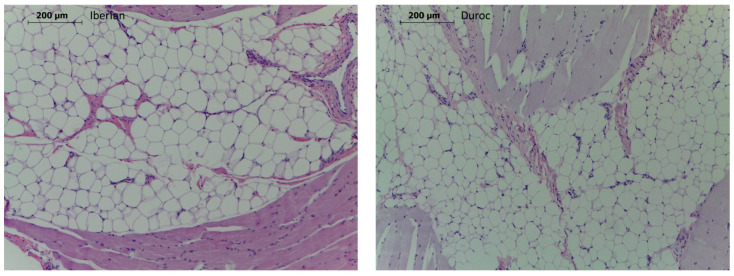
IMF adipocytes of BF muscle of Iberian and Duroc pigs, 91.1 *versus* 78.5 microns, respectively, for these particular samples (photomicrographs taken with a Leica ICC50W™ camera coupled to a Leica DM1000™ microscope).

In the present work, muscle cellularity, composition and meat quality traits have been studied. The size of the IMF adipocytes in the BF muscle was studied in the growing Iberian and Duroc animals, with larger adipocytes being observed in the Iberian breed than in the Duroc breed (86.3 microns *versus* 70.10 microns, respectively; *p*-value < 0.0001). Although there are histological studies comparing the adipocytes of fat and lean breeds [40], to the best of our knowledge, this is the first study that compares, at the histological level, the IMF adipocytes between Iberian and Duroc pure growing pigs. The micrographs of Iberian and Duroc intramuscular adipocytes are shown in Figure 1.

In agreement, a higher IMF content was observed in the BF muscle from Iberian grower pigs than Duroc grower pigs (3.34 *versus* 2.53 g_fat_/100 g_wet product_, respectively; *p*-value = 0.0002). A higher IMF content in Iberian pigs has already been observed when comparing pure IB *versus* IB × Du crossed newborn piglets (2.21 *versus* 1.72, respectively) in BF muscle [24], and in growing pigs (4.05 *versus* 2.87, respectively) in LD muscle [4]. Thus, this quoted work [4] and our present results confirm that the differences in IMF content between Iberian pigs and Duroc genotypes are established at early ages, since similar values have been found in another study with fattened Iberian *versus* IB × Du crossbred animals, also in BF muscle [41]. In Iberian pigs, the adipocytes located between the muscle fibers begin to accumulate lipids from an early age, due to their genetic predisposition for fat accumulation [1,5]. This fact has special relevance at later productive ages, as the high fat content is essential for the organoleptic and quality characteristics of ham dry-cured products. No statistical difference was found for the size of adipocytes nor the IMF content between CH and HO diets.

In this study, meat quality traits related to meat tenderness, such as water holding capacity (thawing and cooking loss percentages), tenderness (shear force), and color parameters (L*, a*, and b*), were determined in Iberian and Duroc growing pigs. Regarding the breed effects on tenderness determinations, the cooking loss percentage and shear force were higher in Duroc pigs and the yellowness (b*) was higher in Iberian pigs. Diet only slightly affected the cooked meat shear force, being higher in animals fed the CH diet, although this difference did not reach the statistical significance threshold (*p*-value = 0.07) (Table 2).

Meat tenderness is the sensory quality trait preferred by consumers. It is influenced by the interaction between genotype, age, diet, and many other factors [42,43,44]. For this reason, the improvement in meat tenderness is receiving increasing attention in pig breeding programs. The Iberian samples had a lower cooking loss percentage and were a more tender meat. These differences between the meat originating from the two breeds can be largely explained by the higher fat deposition and higher IMF content observed in IB pigs [45,46]. Fatty rustic breeds, such as the Iberian breed, have a higher content of oxidative muscle fibers and heme pigment than other selected lean breeds; for example, Duroc. Lindahl et al. (2001) [47] observed a close relationship between heme concentrations in meat and L* and b* values. Thus, a greater amount of heme pigments would lead to higher b* and L* values in Iberian pigs. Moreover, the redness parameter was low in both breeds. This low redness parameter and the lack of differences between breeds were probably due to the age of the animals, both (IB and DU) growing pigs, since the amount of pigments, especially myoglobin, increases with age [10].

FA composition was studied in all the available muscle samples (*n* = 78; 29 Iberian transitional piglets, 30 Iberian growers, and 19 Duroc growers), allowing the evaluation of the effects of age, breed, and diet. The effect of diet on the FA profile of the BF muscle was separately explored in the two age groups. Moreover, age–diet (in Iberian) and breed–diet (in growers) interactions were studied. In all the lipid fractions (NL, PL, and FL), the FA composition showed significant effects on the three main tested factors (Table S3).

Regarding age effects, C18:1n-9 and MUFA content increased with age in all the lipid fractions. Additionally, the stearic and palmitic acid content increased with age in the NL fraction. On the contrary, C18:2n6 and PUFA content decreased with age in the NL and PL fractions, but did not vary in the FL fraction. The higher PUFA and C18:2n6 contents of the NL and PL fractions in transition piglets led to a higher PUFA/SFA and n-6/n-3 ratio, respectively. These results are in agreement with previous studies [48] performed with animals of different pig breeds and similar live weights within the LD muscle [49,50]. Two significant age–diet quantitative interactions were observed in the PL fraction for C18:1n9 and C18:2n6 fatty acids; these interactions are most likely related to the different proportions of FA between the storage (NL) and structural (PL) fractions. The observed increase in MUFA content and the decrease in PUFA content in grower animals, due to the increase in the proportion of C18:1n-9 and the decrease in C18:2n6 fatty acid content, is in agreement with previous observations [11,48].

Breed effects were also found for SFA, MUFA and PUFA concentrations, with higher C16:0, C18:0 and SFA content in the Iberian pigs’ muscle, and higher MUFA and oleic acid in the Duroc pigs’ muscle in both the NL and PL fractions, but not in the FL fraction. The PUFA content was higher in the Duroc pigs in the NL and FL fractions, but not in the PL fraction. These differences, which are similar to those found at the adipose tissue level, were mainly due to a higher content of palmitic and stearic and lower linoleic acid in the Iberian pigs than in Duroc pigs in the NL and FL fractions, but not in the PL fraction. It is also interesting that in the Duroc pigs, the n-6/n-3 ratio was higher in all the lipid fractions studied. This is in concordance with the higher levels of linoleic acid, a precursor of arachidonic acid (C20:4n-6), observed in the Duroc pigs. The main finding regarding FA composition is the higher SFA observed in the Iberian pigs compared to the Duroc pigs, and the unexpectedly higher MUFA content observed in the Duroc pigs (in the NL and PL fractions, but not in the FL fraction). This result is in agreement with the very intense de novo fat synthesis in the Iberian pig tissues, which predominates over FA desaturation in this developmental period, leading to a higher SFA content and a dilution effect for other FAs, such as MUFA and PUFA. On the other hand, the higher levels of PUFA found in the NL and FL fractions in the BF muscle of Duroc pigs, which were also previously observed in the dorsal and ham subcutaneous fat, could be explained by higher ability of the Duroc genotype to store dietary unsaturated lipids in its tissues [7].

It is interesting to remark that the main diet effects on FA composition were detected very early during the treatment, in the first slaughter (7 days of treatment), and moderate changes in the magnitude of differences between the diets were observed along the trial. These results are in agreement with our previous results in a similar diet trial by Ovilo et al., 2014 [51].

Some studies suggested that the diet FA composition led, in general, to a homogeneous response in the different animal tissues [52,53]. However, a previous study in Iberian pigs [54] reported significant effects of diet on backfat and *semimembranosus* muscle, but not on *biceps femoris*. In the present study, the comparison between two diets with different sources of energy (high oleic acid or carbohydrates) showed significant differences in the FA profiles of BF muscle, which, in general, reflected the composition of the diets received, with a higher MUFA content and lower SFA content in the HO group. These differences were mainly due to the higher oleic (C18:1n-9) content in the HO diet. The content of SFA was higher in the CH group, although the CH diet included a lower proportion of palmitic and stearic acids, indicating de novo synthesis of FA from the available carbohydrates in this group. All these results are consistent with the deposition of dietary FA and the induction of lipogenesis by dietary CH. These results are, in general, very similar to those found in our previous work when analyzing the effect of diet on the FA profiles of subcutaneous backfat and subcutaneous ham fat [5,9]. The diet effects observed for SFA and MUFA contents were concordant among the different lipid fractions. However, it is noteworthy that the effects of dietary treatment on C18:2n-6 and total PUFA content were different depending on the lipid fractions. The n-6/n-3 ratio was slightly higher in the CH group in all the fractions in the transition piglets. On the contrary, in the grower pigs, the n-6/n-3 ratio was higher in the HO diet. The NL and PL fractions were the most affected fractions, with the HO diet showing higher C18:1n-9 and C20:1n-9 contents, and lower C18:0 and C16:0 than the CH diet in transition and grower pigs. The observed higher MUFA content in the animals fed the HO diet resulted in a higher MUFA/SFA ratio at both ages. Regarding the PL fraction, a slightly different response was observed, with some quantitative changes in the effect of the dietary supplementation with respect to the NL fraction, due to the different proportions of the two lipid fractions in fatty acids. We observed a higher C18:1n-9 level, but lower C18:2n-6, C18:3n-3 and C18:3n-6 levels, in the PL fraction in the HO group.

Previous works of oleic acid supplementation have been performed in the Iberian breed, focusing on backfat, LD muscle, and liver [51], and on subcutaneous backfat and subcutaneous ham fat adipose tissue in Iberian and Duroc breeds [9]. The present results extend the findings to another tissue, the BF muscle, whose composition is of importance for the production of quality dry-cured ham. According to our results, the employment of an oleic acid-supplemented diet may be a useful tool to improve the quality of cured ham products. This finding is especially interesting in the Duroc breed, which has limited meat quality attributes when compared to the Iberian breed [7,55].

### 3.2. Age, Breed and Diet Effects on the Biceps Femoris Transcriptome

In the present work, RNA-Seq was used in 36 BF muscle samples, in order to study the effect of developmental stage, breed, diet, and interactions on the transcriptome profile in Iberian and Duroc growing pigs. An average of 45 million sequence reads were obtained for each individual sample, and were assembled and mapped to the annotated Sscrofa11.1 genome. All the samples passed the quality control test and 93–95% of the reads were mapped to the porcine reference sequence. An average of 15,314 genes out of 22,452 annotated genes were expressed in the studied samples. Regarding mapping quality values (MAPQ), an average of 96% of the reads showed MAPQ ≥ 30 in the different samples (which corresponds to a probability of a correct match equal to or higher than 0.999).

#### 3.2.1. Changes in Muscle Transcriptome along Growth in Iberian Pigs

The effect of developmental stage on the muscle transcriptome was studied in Iberian pigs, allowing the identification of 1832 DEGs between transitional piglets and growing pigs (FC ≥ 1.5 and BH-adjusted *p*-value < 0.01) (Table S3). We detected 655 DEGs upregulated in the muscle of transition piglets, with FCs ranging from 1.5 to 21, and 1177 DEGs upregulated in growing pigs, with FCs ranging from 1.5 to 23. The genes that showed the largest expression differences according to age were *AHSG* (alpha 2-HS glycoprotein), also known as fetuin-A (FC = 21, *p*-adj = 1.97 × 10^−5^, upregulated in transition piglets), and ENSSSCG00000023048 (FC = 23, *p*-adj = 9.67 × 10^−5^, upregulated in growers), which is a misc_RNA or non-coding RNA. AHSG is a multifunctional molecule that participates in different processes, such as energy expenditure, appetite control, insulin resistance, and regulation of adipogenesis [56].

**Figure 2:**
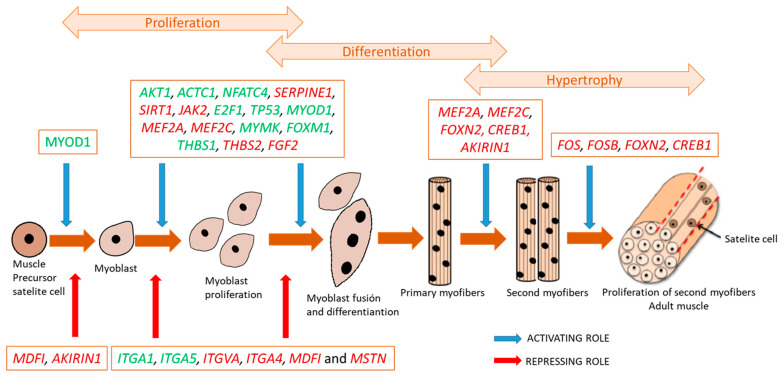
DEGs implicated in myogenesis. Genes in green color are upregulated in transition piglets and genes in red color are upregulated in growers.

Different genes involved in muscle differentiation were upregulated in transition piglets and in grower pigs. The DEGs mainly involved in the early steps of the myogenesis process, such as *MYOD1*, *MYMK*, *AKT1*, and *E2F1*, were upregulated in transition piglets (Figure 2). The *MYOD1* gene (myogenic differentiation 1, FC = 1.81, *p*-adj = 1.14 × 10^−5^) plays a major role in regulating muscle differentiation [17]. *MYMK* (myomaker, myoblast fusion factor, FC = 2.03, *p*-adj = 0.003) regulates the myogenic fusion process [57]. *AKT1* (AKT serine/threonine kinase 1, FC = 1.53, *p*-adj = 0.004) is crucial for myoblast proliferation, but is not necessary for differentiation [58]. *E2F1* (E2F transcription factor 1, FC = 3.06, *p*-adj = 3.13 × 10^−6^) is a member of the E2F family of transcription factors and has a key role in activating primary fiber muscle formation [16]. This gene codes a myogenic differentiation factor that contributes to the induction of terminal differentiation and is essential for postnatal skeletal muscle growth [16]. On the other hand, several DEGs, such as *MEF2A*, *MDFIC*, *MSTN*, *JAK2*, *FOS*, *FOSB* and *AKIRIN1* genes, mostly involved in the formation and proliferation of myofibers and their hypertrophy, were upregulated in growers’ muscles; for example, *MSTN* (myostatin, FC = 1.65, *p*-adj = 0.0009) is a relevant gene that is an inhibitor of skeletal muscle growth [59], and knockout mice show a dramatic increase in muscle mass. *MSTN* upregulation in grower animals may inhibit the over-proliferation of myoblasts when the primary and secondary fibers are forming.

The different patterns of expression of these DEGs along growth seem to be indicative of sequential activation of the different genes involved in the process of skeletal muscle development, as can be observed in Figure 2, with transition piglets showing a deeper activation of genes involved in early differentiation, while growers showed upregulation of further proliferation and later hypertrophy processes.

##### Functional Interpretation

Besides the individual interpretation of selected genes, the total set of DEGs was functionally interpreted. The functional analysis performed with IPA software indicated that the main functional categories, pathways, and regulatory routes in which the DEGs are involved were related to development, cellular growth, cell differentiation, and proliferation. The most enriched functional categories were cellular assembly and organization, cellular function and maintenance, and cell survival and cell viability. The functional analysis showed that the most activated biological function in the transition piglets was morbility or mortality (z-score = −7.835, *p*-value = 1.28 × 10^−18^, with 437 DEGs implicated). The dynamic balance between cell death, proliferation, and differentiation is an important condition for maintaining the development and homeostasis of tissues. Therefore, cell proliferation must be carefully balanced with programmed cell death (apoptosis) to maintain a constant number of cells in adult tissues [60]. Therefore, in transition piglets, the balancing of cell proliferation and cell death may occur in order to carry out the differentiation and growth of tissues in later stages of development. The most activated functions in growers were quantity of cells (z-score = 5.087, *p*-value = 3.56 × 10^−6^, with 238 DEGs implicated), dynamics of the microtubules (z-score = 4.660, *p*-value = 3.14 × 10^−16^, with 234 DEGs implicated), organization of cytoskeleton (z-score = 4.610, *p*-value = 2.55 × 10^−17^, with 270 DEGs implicated), and organization of cytoplasm (z-score = 4.620, *p*-value = 9.61 × 10^−19^, with 298 DEGs implicated) (Table S4).

Sixty-seven canonical pathways were significantly activated or inhibited in the dataset, according to age (*p*-value ≤ 0.01; Table S5). In the transition piglets, five canonical pathways showed activation (z-score < −2, *p*-value ≤ 0.01), such as oxidative phosphorylation and cell cycle control of chromosomal replication signaling pathways, which suggests greater mitochondrial activity in the muscle of young animals with high energy requirements and a very active cell cycle, indicating the occurrence of mainly proliferation and differentiation in muscle cells. On the contrary, sixty-two pathways were significantly activated in the growers (z-score > 2, *p*-value ≤ 0.01). Out of them, twenty-two canonical pathways were related to cell proliferation and differentiation (Table 3 and Table S5), and three of them were related to the organization of the cytoskeleton and ECM, such as signaling by Rho family GTPases, actin cytoskeleton signaling, and JAK/STAT signaling.

**Figure 3:**
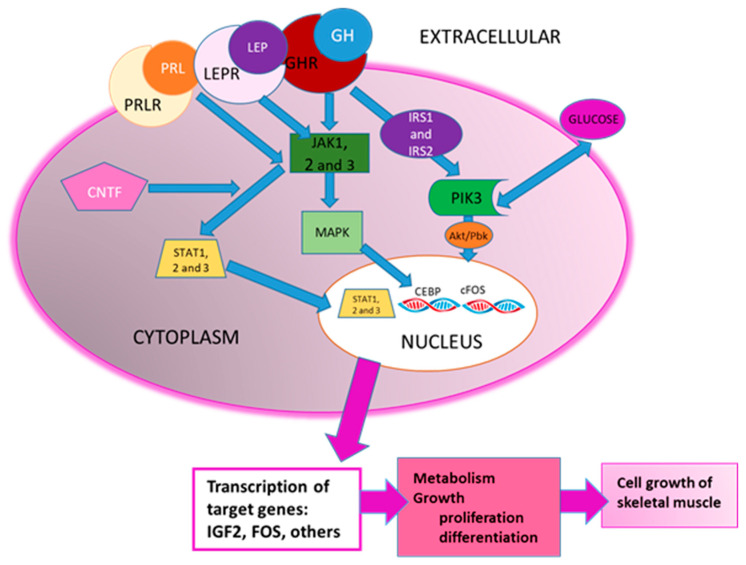
Integrative overview of signaling pathways related to muscle growth and development activated in muscle of grower pigs.

In addition, some of the activated pathways in the grower animals were related to each other, as shown in Figure 3.

The JAK/STAT pathway has a key role in the proliferation and differentiation of muscle cells in mammals [61]. JAKs are a family of non-receptor tyrosine kinases, comprised of JAK1, JAK2, and JAK3. The STATs family is located downstream of JAKs, and includes STAT1, STAT2, and STAT3. The JAK/STAT pathway is activated by ligands, such as cytokines or growth factors (such as growth hormone, prolactin, or ciliary neurotrophic factor), binding to their cognate receptor [61]. This important pathway was found to be activated in grower animals, and, in agreement, the CNTF signaling pathway, which is known to activate the JAK/STAT pathway, was also activated in growers. Similarly, the growth hormone, prolactin and leptin signaling pathways were also activated in growers.

The *oxidative phosphorylation pathway* has the highest activation, z-score = −4.243, detected in transition piglets (Table 3). Oxidative phosphorylation (OxPhos) takes place inside the mitochondria, and is essential to modulate cell metabolism and energy homeostasis, due to its key functions in energy retention, generation of reactive oxygen species (ROS), anabolism and iron catabolism, calcium and iron homeostasis, apoptosis, and signal transduction. Mitochondria are, therefore, critical organelles responsible for regulating the metabolic state of skeletal muscle [62,63,64], which, moreover, is the tissue, together with cardiac muscle, with the highest concentration of mitochondria [65,66]. Moreover, it is also known that in young animals, oxidative fibers predominate in the muscle, and these fibers have a greater number of mitochondria [67]. This preponderance of oxidative fibers may contribute to better mitochondrial homeostasis and a greater capacity to maintain mitochondrial function in transition piglets. In addition, recent studies have indicated that mitochondria play a key role in the regulation of myogenesis, and this is associated with a change in metabolism from glycolysis to OxPhos as the major energy source for mitochondrial enzyme activity [68]. On the other hand, it is known that *MYOD1* (upregulated in transition piglets) is important for postnatal myoblasts to progress through differentiation to form adult skeletal muscle [69]. Furthermore, changes in mitochondrial number and activity, as well as mitochondrial dysfunction, are implicated in aging and age-related diseases, increased oxidative damage, decreased mitochondrial quality, and reduced activity of metabolic enzymes [70,71]. Thus, in transition piglets, the activation of oxidative phosphorylation may be involved in the regulation of myogenesis, and the enhancement of energy and cellular homeostasis.

##### Upstream Regulator Analysis

The upstream analysis (regulator and causal network prediction) and regulator effects tools of the IPA package were employed to identify potential transcriptional regulators that may explain the differential patterns of expression observed between ages, and allowed the identification of 333 candidate regulators (*p*-value < 0.05; Table S6 and Table 4).

Moreover, the sense of activation state was predicted for some of the identified regulators. In the transition piglets, 37 upstream regulators were activated (z-score < −2), mainly related to cell growth, proliferation, and differentiation. On the other hand, 28 upstream regulators were activated in the growing animals (z-score > 2, Table S6 and Table 4).

**Figure 4:**
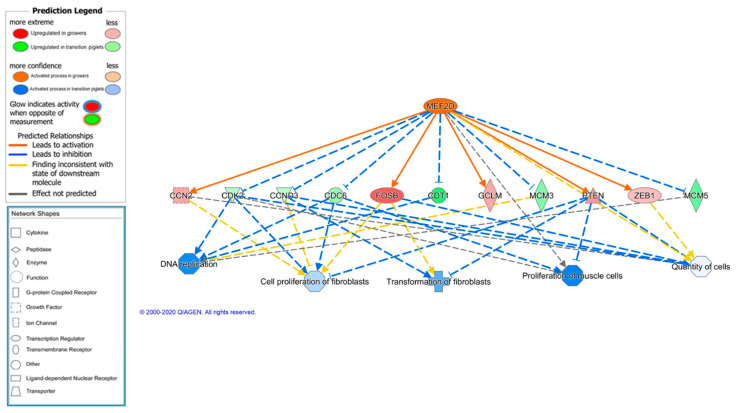
Causal network of MEF2D regulator, which is activated in growers and leads to predicted inhibition of several functions related to proliferation in growers (activated in transition piglets) (z-score = 3.317, *p*-value = 0.002).

The myocyte enhancer factor 2 (MEF2) is an important transcription factor involved in the development of skeletal muscle, since it plays a key role in the differentiation of muscle cells [16]. In differentiated myotubes, four isoforms of MEF2 (A–D) have been identified [72], and all but MEF2B are reported as being expressed in skeletal muscle. In our study, the isoforms MEF2A and MEF2C were upregulated in the growers (FC = 1.82, *p*-adj = 0.0004 and FC = 1.45, *p*-adj = 0.03, respectively). Moreover, the MEF2D isoform was predicted to be an activated upstream regulator in the growing animals (z-score = 3.238, *p*-value = 0.0001) and is also responsible for a causal network (z-score = 3.317, *p*-value = 0.002; Figure 4). In this causal network, MEF2D activates several upregulated genes (red nodes) and inhibits other downregulated genes (green nodes) in growers, leading to the inhibition of several relevant functions related to proliferation in the growers (blue nodes, predicted to be activated in transition piglets). Furthermore, it is known that MEF2 activation in skeletal muscle is regulated through several parallel intracellular signaling pathways, in response to insulin or cellular stress, such as the AMPK signaling pathway. In addition, the p38 MAPK pathway also promotes skeletal muscle differentiation, at least in part, through the activation of MEF2 [72,73,74]. In agreement, these two pathways (AMPK and p38 MAPK signaling pathways) are also activated in grower animals (Table S5 and Table 3). The joint results agree with a more proliferative tissue in transition piglets and a more advanced muscle differentiation stage in the growers, and provide information on the key molecules regulating this progression in muscle development.

**Figure 5:**
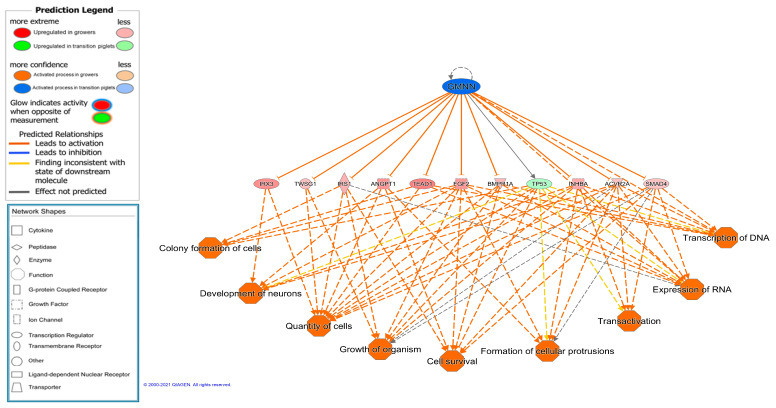
Regulator effects network predicted as activated in transition piglets, controlled by GMNN regulator.

Several regulator effects networks were also identified. Interestingly, a network was identified and predicted to be activated in transition piglets, which was involved in nine activated functions in the growers, related to growth (Figure 5). The gene expression of ten DEGs upregulated in growers is controlled by the geminin (GMNN) regulator (activated in transition piglets), which has a central function in regulating the cell cycle and differentiation [75].

**Figure 6:**
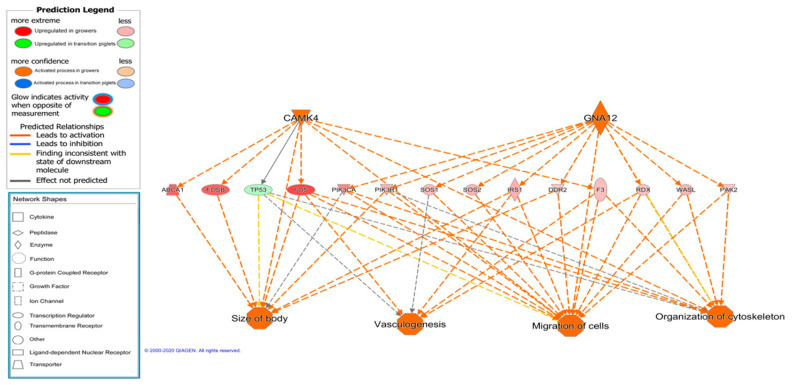
Regulator effects network predicted to be activated in growers, controlled by CAMK4 and GNA12 regulators.

In addition, another interesting regulatory network was identified and predicted to be activated in growers, which is involved in the size of the body, vasculogenesis, migration of cells, and organization of the cytoskeleton, and includes the transcriptional regulators CAMK4 and GNA12 (Figure 6). CAMK4 calcium/calmodulin-dependent protein kinase operates in the calcium-triggered CaMKK–CaMK4 signaling cascade and regulates, mainly by phosphorylation, the activity of several transcription activators, such as MEF2D, which is implicated in muscle growth and development, and activated in grower animals, and was discussed previously [76]. GNA12 (guanine nucleotide-binding protein 12) is involved, as a modulator or transducer, in various transmembrane signaling systems, and GNA12-dependent Rho signaling subsequently regulates the FOS transcription factor (upregulated in grower animals; FC = 3.75, *p*-adj = 0.0001), which is implicated in cell differentiation and transformation [77].

Skeletal muscle development and growth are complex processes, highly organized and regulated by the interaction of muscle cells with their extracellular microenvironment. These processes require specific cytoskeletal, membrane and adhesion proteins. Our results of functional enrichment and activation (Table S4) showed relevant functions activated in growers; these functions are directly involved in the organization of the cytoskeleton and cytoplasm, cell quantity and adhesion, and the dynamics of the microtubules, which are functions closely related to extracellular matrix (ECM) organization. Moreover, three activated pathways in the grower animals (ephrin receptor signaling, signaling by Rho family GTPases, and actin cytoskeleton signaling) (Table S5 and Table 3) were also related to cytoskeleton and ECM organization.

The cytoskeleton is composed of a polymeric arrangement of proteins and structures organized in the following three specific systems: microfilaments, intermediate filaments, and microtubules [78,79]. All these components have to change during myogenesis to accommodate the physiological muscular adaptation. They are also highly integrated and, in turn, interact mechanically with the ECM. Microtubules are major dynamic structural components of the cytoskeleton, and have several roles in myogenesis and in a variety of cell processes [80]. Microtubules are polymers of α and β tubulin proteins. Tubulin is encoded by a multigene family that produces a distinct set of gene products or isotypes [79,81]. The changes in cell shape during myogenesis are caused by the rearrangement of microtubules, which are extensively remodeled, and their radial distribution is changed by their longitudinal distribution in the elongated myotubes. Because of these dynamics and their mechanical properties, microtubules take part in various essential processes, from intracellular transport to the search and capture of chromosomes during mitosis [82]. In this study, the organization of the cytoskeleton and the dynamics of the microtubules were the most activated functions in the grower animals, with a high number of DEGs involved, such as kinesins (*KIF2A*, *KIF3A*, *KIF5B*, *KIF12*, etc.), myosins (*MYO6*), dyneins (*DYNLT3)*, centrosomal proteins (*CEP57*, *CEP290*, *CEP350*, etc.), coiled-coil domain-containing proteins (*CCDC8*, *CCDC14*, *CDCD57*, etc.), and others [83]; all of these DEGs were upregulated in the grower animals. The increase in the organization of the cytoskeleton along growth is correlated with an increase in cell size (hypertrophy), since there is greater growth in grower animals [84]. Additionally, cytoskeletal dynamics are controlled by the Rho GTPases signaling pathway activated in grower animals, which mediates the signaling between several membrane receptors, such as tyrosine kinases (*JAK2*) and integrins (*ITGVA* and *ITGA4*), and is also upregulated in growers. However, intriguingly, several α and β tubulins were upregulated in the transition piglets (*TUBA1B*, *TUBA1C*, *TUBA4A*, *TUBB*, and *TUBB6*), most likely suggesting an increase in microtubule networks, leading to greater cell division and proliferation in the piglets, providing muscle cells for adhesion and later differentiation [85].

The ECM of skeletal muscle is a dynamic mixture that has both mechanical support and active signaling roles [86]. The ECM is composed of a great variety of structural glycoproteins and polysaccharide molecules assembled in a molecular network. These components interact with other cells, such as fibroblasts, adipocytes, or immune cells [87]. Moreover, the ECM and the connective tissue surrounding skeletal muscles regulate muscle development, growth, and repair through their communication with muscle cells [88]. These interactions are mediated by different transmembrane molecules (collagens, laminins, actins, myosins, and integrins), and these components drive the direct or indirect control of cellular activities, such as differentiation and proliferation. When cell proliferation is increased, it provides a larger pool of muscle cells available for further differentiation and adhesion [87]. Integrins are the main receptors responsible for the attachment of cells to the ECM. Integrins also interact with components of the cytoskeleton to provide a stable bond between the ECM and adherent cells. In addition to this structural function, integrins serve as receptors that activate intracellular signaling pathways [60]. Several integrins were differentially expressed according to our results, such as *ITGA1* and *ITGA5,* which were positively regulated in the transition piglets. Moreover, *ITGAV* and *ITGA4* were positively regulated in the growers, which suggests sequential activation of the different integrins involved in the process of skeletal muscle development. Collagens and laminins are found abundantly in the ECM environment, and are vitally important for the mechanical support of tissues, in addition to cell adhesion and differentiation [89]. Several collagens were differentially expressed along growth, such as *COL4A3*, *COL6A6*, *COL8A1*, and *COL17A1*, which were upregulated in the growers, and *COL20A1* was upregulated in the transition piglets. Additionally, the laminin *LAMA2* was upregulated in the growers. This laminin is a major component of the basement membrane, and is thought to mediate the attachment, migration, and organization of cells into tissues during embryonic development by interacting with other ECM components [90]. The ECM proteins involved in myoblast differentiation act by regulating the interaction of myostatin (*MSTN*) with its receptor, activin receptor type IIA (*ACVR2A*) [91], both of which are upregulated in grower animals (FC = 1.65, *p*-adj = 0.0009 and FC = 1.57, *p*-adj = 0.005, respectively).

Altogether, our results of differential expression and functional interpretation between growing stages are compatible with the transition piglets showing preponderance of early differentiation events (from satellite cells to myoblast), and also proliferation and hyperplasic processes, while the grower animals show preponderance of differentiation of muscle cells, leading to hypertrophy and growth of skeletal muscle.

#### 3.2.2. Breed Effects on the Transcriptome of Grower Pigs

DESeq2 identified 1055 annotated DEGs between breeds (FC ≥ 1.5 and BH-adjusted *p*-value < 0.05), of which 505 genes were overexpressed in IB pigs (FC ranging from 1.5 to 56) and 550 DEGs were overexpressed in DU (FC ranging from 1.5 to 15) (Table S7). The genes showing the largest expression differences between breeds were *CLCA1* (chloride channel accessory 1, FC = 56, *p*-adj = 1.34 × 10^−12^ overexpressed in IB) and *PCK2* (phosphoenolpyruvate carboxykinase 2, mitochondrial, FC = 15, *p*-adj = 1.67 × 10^−9^ overexpressed in DU). The *CLCA1* gene is thought to act as a signaling molecule for the innate immune response, through the activation of macrophages, and, consequently, increases pro-inflammatory cytokine release (IL-8, IL-6, IL-1, and TNF-α). Additionally, it has relevance in the early innate immune response in murine models [92]. Interestingly, this gene was also detected as overexpressed in Iberians, showing the highest expression difference between breeds, in the previous study of the transcriptome of subcutaneous ham adipose tissue in the same experimental animals employed here [5], indicating consistent regulation across tissues. The *PCK2* gene encodes a mitochondrial enzyme that catalyzes the conversion of oxaloacetate to phosphoenolpyruvate in the presence of guanosine triphosphate (GTP) [93]. In addition, *PCK2* may be associated with muscle anabolism and cell proliferation [94]. A cytosolic form of this protein is encoded by the *PCK1* gene and has also been associated with meat quality traits in pigs [95].

In Iberian pigs, overexpressed genes with known biological functions related to lipid and protein metabolism and energy homeostasis, such as *ME1*, *FASN*, *G0S2*, and *PDK4*, have been identified in the present study, which is in agreement with the breed’s adipogenic trend. The malic enzyme (*ME1*, FC = 1.62, *p*-adj = 0.0009) gene is involved in fatty acid synthesis, encoding a cytosolic enzyme that produces NADPH (dihydronicotinamide adenine dinucleotide phosphate) [96,97]. Higher expression of the *ME1* gene was also observed in our previous results obtained by qPCR and RNAseq from subcutaneous fat samples [5,9,98]. The fatty acid synthase (*FASN*) (FC = 1.99, *p*-adj = 0.03) is a central enzyme for fatty acid synthesis, which incorporates acetyl-CoA and malonyl-CoA, derived from glucose or other carbon precursors, to form palmitate [99]. The G0/G1 switch 2 (*G0S2*) (FC = 1.51, *p*-adj = 0.05) gene is a negative regulator of lipolysis, whose activation is known to downregulate *ATGL* expression. There is evidence that illustrates the critical roles of this gene in modulating energy metabolism, cell growth, and apoptosis in a multitude of cell types [100]. The mitochondrial multienzyme complex pyruvate dehydrogenase (PDC) catalyzes pyruvate decarboxylation to acetyl-CoA, which modulates the harmonization between glucose and lipid oxidation in mammals [101]. In particular, *PDK4* is overexpressed in Iberian pigs (FC = 2.38, *p*-adj = 0.02), controlling the activity of PDC in skeletal muscle, and contributes to glucose and FA metabolism (FA oxidation and de novo FA biosynthesis) regulation [102]. In fact, this gene is considered to be a candidate gene for fattening traits, and its structural variation has been associated with IMF and muscle water content and other meat quality traits in pigs [103]. This gene was also found to be upregulated in the fatty Mangalitsa breed in a comparison between the muscle transcriptomes of Mangalitsa and Moravka pig breeds [104]. These results are concordant with the known high lipogenesis potential of fat pig breeds, such as the Iberian [1,105].

Among the genes overexpressed in Duroc pigs, and coinciding with our results in ham subcutaneous adipose tissue [9], we found the *IGF2* gene (FC = 3.03 and FC = 2.67, *p*-adj = 1.19 × 10^−5^ and *p*-adj = 2.18 × 10^−18^, in muscle and adipose tissue, respectively), which encodes for a member of the insulin family of polypeptide growth factors, which participate in growth and cell differentiation. *IGF2* stimulates the proliferation and differentiation of myoblast and satellite cells. Moreover, it is a paternally imprinted gene, linked to heart size, fatness, and muscle growth. In swine, a causal mutation in *IGF2* intron 3 has been identified (g.3072G > A) [106,107]. This mutation influences production and carcass characteristics, and displays different alleles in Duroc and Iberian breeds. A high frequency of the mutant IGF2 g.3072A allele is observed in the Duroc breed, while it is almost absent in Iberian pigs [108,109]. The higher postnatal *IGF2* expression is associated with the mutation [108], and, thus, the overexpression of *IGF2* in Duroc pigs is in concordance with previous observations and with the presence of alternative alleles in both the studied breeds.

It is interesting to note that the *DLK1* gene was found among the DEGs overexpressed in the Duroc pigs (FC = 2.98, *p*-adj = 1.3 × 10^−7^). *DLK1* is expressed in preadipocytes, but is absent in mature adipocytes [110], suggesting a higher content of preadipocytes in DU pigs and possibly a higher content of mature adipocytes in IB animals. This interesting result agrees with the results found in crossbred animals, where a higher content of preadipocytes in DU x IB animals’ vs. a higher content of mature adipocytes in pure Iberian pigs was found, and, thus, adipogenesis occurs earlier in the latter [4]. This also agrees with the difference in adipocyte size observed at the histological level. On the other hand, it supports the hypothesis of a higher lipogenic capacity in the muscle of pure IB animals, which is reflected at the transcriptome level.

##### Functional Interpretation

Following gene ontology (GO) enrichment, 299 biological functions were predicted to be affected by breed (*p*-value < 0.01) (Table S8) and twenty-nine canonical pathways were significantly enriched (*p*-value < 0.1) in the dataset of 1055 DEGs (Table S9), with six pathways being predicted to be significantly activated or inhibited (z-score > 2 or <−2) (Table 5).

The functions activated (z-score < −2) or enriched (z-score with a negative sign) in the Iberian breed, and the activated pathways, were mainly related to development and growth, cytoskeleton organization, and adipogenesis, such as development of the body trunk (z-score = −2.215, *p*-value = 6.32 × 10^−6^), development of vasculature (z-score = −1.63, *p*-value = 6.56 × 10^−7^), quantity of cells (z-score = −1.770, *p*-value = 5.57 × 10^−14^), organization of the cytoskeleton (z-score = −1.862, *p*-value= 7.73 × 10^−6^), and microtubule dynamics (z-score = −1.848, *p*-value = 1.83 × 10^−5^) functions, and *growth hormone* and *GM-CSF* signaling pathways (Tables S8 and S9 and Table 5). These pathways and functions involved several DEGs overexpressed in IB pigs, such as *JAK2*, *FOS*, *MAPK4*, *STAT1*, *IRS2*, and *GHR*, among others. Interestingly, the two breed groups did not differ in body weight at the end of the experimental period (51.2 kg vs. 50.2 kg in the Duroc and Iberian pigs, respectively, *p*-value = 0.6). This is in agreement with the findings reported by Ayuso et al., 2016 [4], who also found no difference between purebred Iberian and Duroc crossbreeds (IB × Du) in the growing period, after a smaller birth weight was observed in the purebred animals, indicating catch-up growth in the early growing stages of Iberian animals, which is compatible with the activation of growth functions and pathways in the muscle transcriptome of Iberian animals reported here. Moreover, several pathways with suggestive activation in Iberian pig muscle include adrenomedulling signaling, related to angiogenesis and resilience after oxidative stress and hypoxic injury [111], and the thrombopoietin signaling pathway, related to proliferation, differentiation, and cell survival [112]. Thus, these findings also support the improved viability of Iberian pig muscle, supporting the hypothesis of a compensatory gain experienced in Iberian pigs in early growth.

Regarding FA metabolism-related functions, in the Iberian fatty breed, we observed that the concentration of triacylglycerol function and the triacylglycerol biosynthesis pathway were enriched (z-score = −1.000, *p*-value = 0.1). Triacylglycerols are the main form of fat storage in the tissues, and are also responsible for thermogenesis [14]. The Iberian breed, due to its thrifty genotype, has a tendency to accumulate fat to be used in times of low availability of food and adverse environmental conditions [24]. Moreover, in this breed, we observed enrichment in eicosanoid metabolism function (z-score = −1.133, *p*-value = 5.63 × 10^−5^), indicating greater catalytic oxidation of fatty acids.

On the contrary, the synthesis and metabolism of acylglycerol function (z-score = 2.191, *p*-value = 5.36 × 10^−5^) (Table S8) was activated in the Duroc breed, as well as the triacylglycerol degradation pathway (z-score = 1.633, *p*-value = 0.02) (Table S9 and Table 5). These processes seem to indicate intense metabolic activity (synthetic, but also catabolic) of the lipids in the Duroc BF muscle, which would result in lower net lipid deposition in comparison to the Iberian pig muscle, and is in agreement with the lower intramuscular fat deposition observed for the Duroc animals with respect to the Iberian animals. These processes may also be favored by the greater insulin sensitivity (z-score = 1.536, *p*-value = 1.76 × 10^−6^) and glucose tolerance (z-score = 1.178, *p*-value = 1.65 × 10^−5^), which are also activated functions in the Duroc breed [113,114].

Two pathways activated in the Duroc breed (the *retinol biosynthesis* and *LXR/RXR* activation signaling pathways) (Table S9 and Table 5) are intimately related to each other. Retinoic acid acts as a ligand for the family of nuclear retinoid X receptors (RXR), and these receptors regulate the expression of genes involved in tissue development, cell differentiation, and cellular homeostasis [115]. In addition, the LXR/RXR activation pathway is involved in the regulation of lipid metabolism, inflammation, and cholesterol-to-bile acid catabolism [8,9,10,11,12,13,14,15,16,17,18,19,20,21,22,23,24].

##### Upstream Regulator Analysis

An in silico prediction of potential transcriptional regulators allowed the identification of 73 regulators or molecules that may explain the differential expression patterns observed between the breeds (*p*-value < 0.01; Table S10 and Table 6). In Iberian pigs, twelve regulators were activated (z-score < −2), mainly related to cell growth, such as NUPR1, FOXO3, PDGF BB, Rb, and HDAC2, or related to carbohydrate and lipid metabolism, such as HDL cholesterol, IB3, FOXO3, ICAM1, and ALDH2. On the other hand, in the Duroc pigs, seven regulators were activated (z-score > 2), mainly related to development and growth, such as MITF, IGF2BP1, and RABL6, or related to ECM organization, such as COL6A1 and COLQ.

**Figure 7:**
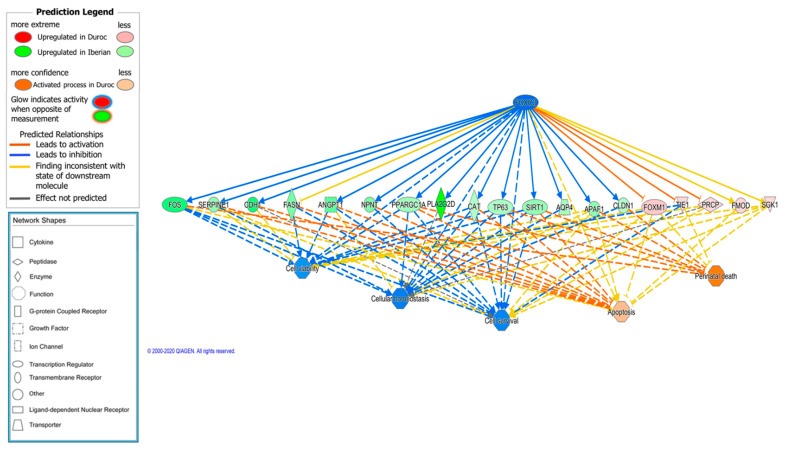
Causal network of FOXO3 regulator activated in Iberian pigs (z-score = −2.982; *p*-value 3.7 × 10^−5^).

We found twenty activated regulators in the Iberian breed. Among them, a causal network was predicted for the FOXO3 transcription factor, which is activated in Iberian pigs and regulates the expression of a diverse array of genes involved in redox homeostasis, cell proliferation, differentiation, metabolism, and apoptosis [116]. This regulator has important roles in muscle hypertrophy and atrophy, and is also activated in the LD muscle of Iberian fetuses when compared with Large White crossbreeds [114] (Figure 7).

Most of the differences found between the DU and IB pigs, in functions and pathways (Tables S8 and S9 and Table 5), were involved in muscle development and growth, and these differences could be useful to understand the genetic and transcriptional mechanisms underlying muscle development. Relevant functions related to cytoskeleton organization and microtubule dynamics were activated in the IB pigs (Table S8). As explained before for the age effect, cytoskeleton organization has a key role in myogenesis. Moreover, the cytoskeleton and ECM are closely related, both being involved in muscle integrity, growth, and fat deposition, as well as muscle connective tissue content [78]. The following three integrins were differentially expressed between breeds: *ITGA2* (FC = 2.05, *p*-adj = 0.001, upregulated in DU), and *ITGA4* and *ITGA8* (FC = 1.88 and FC = 1.71, *p*-adj = 0.04 and 0.001, respectively, upregulated in IB). It has been proposed that integrins also regulate angiogenesis [117]. In fact, functional enrichment analysis (Table S8) has shown angiogenesis and development of the vasculature as enriched functions in the Iberian breed (z-score = −1.063, *p*-value = 1.5 × 10^−5^, and z-score = −1.063, *p*-value = 5.6 × 10^−7^, respectively), with important genes, such as *ANGPT1* (angiopoietin 1, FC = 2.20, *p*-adj = 0.002), overexpressed in this breed. There is evidence of the close relationship between the development of adipocytes and vasculature; hence, the influence of integrins on angiogenesis may have an impact on adipogenesis as well. In fact, adipogenesis and angiogenesis are reciprocally regulated [89,118], and the differentiation of adipocytes was also an enriched function in the IB breed (z-score = −1.69, *p*-value = 3.1 × 10^−7^), with *CEBPD*, a key transcriptional regulator of adipogenesis, also overexpressed in the IB breed (FC = 1.77, *p*-adj = 0.003). Moreover, in the muscle, ITGAs bind to collagens and both types of molecules interact in the maintenance of the ECM [119]. Specifically, *ITGA2*, which is overexpressed in DU, has collagens as ligands and five different collagen genes were also overexpressed in the DU pigs (*COL6A6*, *COL8A2*, *COL11A1*, *COL11A2*, and *COL20A1*), and one collagen (*COL17A1*) was overexpressed in the IB pigs. In addition, other ECM-related proteins, such as *FBN2* (fibrillin 2, FC = 1.55 and *p*-adj = 0.01) and *FMOD* (fibromodulin, FC = 1.60 and *p*-adj = 0.04), were overexpressed in the DU pigs. The results of *ITGA2* and collagen expression are in agreement with our findings in a previous study in crossbred DU × IB pigs [8], although the specific regulated collagens are not coincident in both works, probably due to the fact that the previous work employed another muscle (LD) and recently weaned animals. Muscle collagen content can affect the toughness of meat, influence tenderness, and is associated with growth rate [120]. Moreover, collagen development is negatively correlated to adipocyte development in the ECM; if too much collagen is deposited, the growth of adipocytes is hindered, and, conversely, the removal of collagen stimulates the metabolism and survival of adipocytes [121]. The greater predisposition of the Iberian breed to accumulate fat in their adipocytes, and their larger size, is in agreement with the higher content of collagen in the Duroc breed, hindering the growth of their adipocytes and the accumulation of fat. The higher collagen expression observed in the DU muscle is also in agreement with the observed lower meat tenderness (higher cooking loss and shear force), and lower IMF and adipocyte size in comparison to IB. Further, collagen deposition in intramuscular locations starts at an early developmental stage [122,123], and could be predictive of collagen deposition in later stages. Therefore, these results suggest that the higher collagen gene expression observed in the growing DU muscle is also in agreement with the reported higher lean growth, but lower meat tenderness and intramuscular fat content, of fattened pigs [124].

The transport of materials across membranes is a vital process for all aspects of cellular function, including growth, metabolism, and communication between cells [125]. Protein transporters are the molecular gates that control this movement and serve as key points of regulation for these processes. Solute carriers (SLCs) represent the largest family of protein transporters. Despite the importance of these proteins in determining metabolic states and adaptation to environmental changes, a large proportion of them are still orphan and lack associated substrates [125,126]. In this study, a large number of SLCs (Table S7) were differentially expressed between the breeds, most of them being overexpressed in the Duroc breed, indicating that the transport of solutes in the muscle cell in each breed is very different.

Muscle mass markedly increases along postnatal development, through the process of hypertrophy that takes place when the overall rates of protein synthesis exceed the rates of protein degradation, leading to protein accretion. The whole-body protein turnover and protein synthesis/protein deposition ratio differ between Iberian and other lean breeds, such as Landrace [127,128]. It has been reported that the Iberian breed has limited capability for protein accretion at maximal growth compared to lean pig breeds and their crosses [129]. This fact has been previously associated with higher expression of protein catabolism genes in muscle transcriptome studies [4,5,6,7,8]. In agreement with these previous findings, some genes related to protein degradation were overexpressed in the Iberian muscle, such as *NOS1*, *CTSL*, *TRIM23*, and *TRIM24* (FC = 1.51, FC = 1.93, FC = 1.57, and FC = 1.58, respectively). *NOS1* and *CTSL* differential expression was consistent with previous findings, but different types of *TRIM* genes were differentially expressed in comparison to previous work [8].

##### Joint Interpretation of Age and Breed Effects on Muscle Transcriptome

It is interesting to note that out of the 1832 DEGs affected by age in Iberian growing pigs and 1055 DEGs affected by breed at the growing stage, as many as 397 DEGs were common (Table S11), which were affected by both age and breed. Remarkably, out of 397 common DEGs, the common DEGs overexpressed in transition piglets (*n* = 190) were all coincident to the DEGs overexpressed in the DU breed, and the common DEGs overexpressed in the growers (*n* = 207) were all coincident to the DEGs overexpressed in the IB pigs. Many of these common genes were closely related to muscle development and growth, such as the *MYOD1*, *MYBL2*, *FOS*, *JAK2*, *E2F1*, *FOXM1*, *MSTN* and *DLK1* genes, and were also related to the organization of the cytoskeleton and microtubule dynamics, such as *ATXN1, CDC20*, *JAK2, KAT2B*, and several kinesin-like genes; for instance, our data show higher expression of myostatin (*MSTN*) in the grower animals in comparison to the transition piglets (FC = 1.65, *p*-adj = 0.0009), and higher expression in the IB pigs in comparison to the DU pigs (FC = 1.38, *p*-adj = 0.06). On the other hand, *MYOD1* is an example of the opposite situation, being overexpressed in the transition piglets (FC = 1.81, *p*-adj = 1.41 × 10^−7^) and in the DU pigs (FC = 1.36, *p*-adj = 0.08). Other relevant genes involved in the early proliferative processes of muscle, such as the *MYBL2, E2F1, FOXM1, DLK1* and *NFATC4* genes, followed this same pattern of expression, being overexpressed in the transition piglets and in the DU animals. On the other hand, key genes in the processes of differentiation and hypertrophy, such as *JAK2, FOS, GHR, FOXN3,* and *CTF1*, followed the contrary pattern of expression, being overexpressed in the grower pigs and in the IB animals, indicating that these animals could be in a more advanced stage of muscle development. The differential expression pattern of all the common genes, affected by age and breed, would support the hypothesis stating that both breeds progress with different paucity regarding muscle development, with the Iberian breed being in a more advanced stage of development than the Duroc breed, or, in other words, that the Iberian breed is more precocious in muscle development than the Duroc breed, at this specific growing stage. Similar hypotheses have been postulated, proposing that myogenesis starts earlier, but progresses more slowly, in local fat pigs than in lean pigs [16,17].

#### 3.2.3. Diet Effects on Muscle Transcriptome

The diets employed in the present study were formulated to represent a standard pig diet with carbohydrates as the energy source (CH diet), and an alternative diet, including high oleic sunflower oil (HO diet), which is employed in Iberian pig intensive production, to mimic the traditional extensive production system in which animals are fed acorns, rich in oleic acid, and grass. The diets were isocaloric and isoproteic, and, thus, they differed in several ingredients besides oleic acid, such as fiber and SFA. Therefore, it is not possible to study the isolated effect of one particular nutrient, but the aim of this study was to be as close to the practical scenario as possible and deepen the understanding of the molecular events differing in the animals fed a standard carbohydrate diet vs. a high oleic acid isocaloric diet. The effect of diet on the muscle transcriptome was studied in the two development stages in the Iberian animals. In addition, the effect of diet was also studied in the Duroc growing animals. We detected eight DEGs that were conditional on diet in the transition Iberian piglets and eight DEGs that were conditional on diet in the grower Iberian animals. No common genes were found between both breeds. In addition, three DEGS that were conditional on diet were detected in the growing Duroc pigs (Table S12 and Table 7). One common DEG was observed between the two growing breed groups (*MYL4* gene), although the differential expression was opposite in the two breeds (upregulated in the HO diet in the Iberian pigs and in the CH diet in the Duroc pigs).

The effect of diet on the muscle transcriptome was smaller than the effect observed in these same experimental animals on the subcutaneous ham adipose tissue, with only a few genes affected by diet at both development stages in the Iberian animals and in the Duroc growing pigs. This lower responsiveness of muscle tissue to diet interventions, at the gene expression level, was also observed in previous studies focused on candidate genes in animals fed different diets [51,130,131]. The very scarce number of DEGs observed did not allow a powerful functional analysis of the results to be performed. However, despite this low number of DEGs, enrichment of the biological functions related to lipid metabolism and cell signaling was detected in the transition Iberian piglets, and functions related to the cellular cycle, cellular assembly, and organismal development were enriched in the grower Iberian animals. In the Duroc pigs, we found enrichment of the functions related to carbohydrate metabolism, energy production, and organismal and tissue development.

#### 3.2.4. Interactions Effects

In order to deepen the potential interaction effects, we used the DESeq2 tool to perform a joint analysis using a full model, fitting the age–diet and breed–diet interactions (Table S13). This analysis yielded the following three genes with significant age–diet interaction effects (*p*-adj < 0.1): *NEFH, LY6G6C*, and *SCN10A*. The first gene codes a neurofilament heavy polypeptide that is a structural constituent of the cytoskeleton, and is related to cytoskeleton organization, the transmission of nerve impulses, and axon development [132]. The second gene is a member of the lymphocyte antigen 6 family, belonging to a cluster of leukocyte antigen 6 genes located in the major histocompatibility complex class III, which have definite or putative immune-related roles [133]. The last member is a sodium voltage-gated channel alpha subunit 10, and it is related to the transport of sodium ions and the perception of pain [134]. All of the members present greater expression in the CH diet in the transition piglets and in the HO diet in the grower animals.

This analysis also yielded the following three genes with significant breed–diet interaction effects (*p*-adj < 0.1): *MYL4, MYL5,* and *CD36*. Myosins are involved in several functions, such as muscle contraction, cytokinesis, cargo transport, cell adhesion and migration, and the formation and stabilization of actin-rich structures. *MYL4* is involved in muscle filament sliding and in the positive regulation of ATPase activity. The *MYL5* gene encodes one of the myosin light chains, a component of the hexameric ATPase cellular motor protein myosin [135]. *CD36* appears to be a food-sensitive lipid sensor in the gustatory circumvallate papillae, although it is not only expressed in the taste buds, but also in other tissues. Lipid-regulated alteration in lingual *CD36* expression might modify the interest for fat during a meal, which would initially be high and would then progressively decrease after feed intake. This lipid-mediated regulation is reminiscent of sensory-specific satiety. Our findings apparently support the role played by *CD36* in the oro-sensory perception of dietary lipids [136,137]. The three quoted genes showed greater expression in the CH diet in the Duroc animals and in the HO diet in the Iberian animals. These interactions are intriguing and difficult to interpret, thus future research is needed to deepen their understanding and implications.

#### 3.2.5. Results Validation by Quantitative PCR (qPCR)

With the purpose of validating the reported observations from the RNA-Seq analysis, we selected 12 representative genes, and their relative expression was analyzed by qPCR in all the studied samples (*n* = 36). Selection was performed from the lists of DEGs for the breed and age effects. The selected DEGs were technically validated, showing significant results in the correlation between the RNA-Seq and qPCR results (Table 8, correlation values ranging from 0.62 to 0.94 and p-values ranging from 0.01 to 1.84 × 10^−11^). In order to assess the technical validation in high-throughput transcriptomic studies, we also calculated the CCC coefficient, and it is interesting to highlight that a value of 0.80 was obtained, which supports a considerable general relationship between the RNA-Seq and qPCR expression values [35]. Regarding the age effect in Iberian pigs, eight out of twelve DEGs, selected according to RNA-Seq, were also significant after qPCR analysis, and for the breed effect, eight out of twelve DEGs, detected with RNA-Seq, had confirmed significance in the qPCR analysis (Table 8).

## 4. Conclusions

We found a strong effect of age and considerable effect of breed on the muscle transcriptome. Nevertheless, the effect at the transcriptome level of the two employed diets at this particular developmental stage was small. Most differences in the transcriptome profiles between the transition Iberian piglets and grower Iberian animals, and between the Iberian and Duroc growing pigs, were related to growth, muscle development, and cytoskeleton and ECM organization. In addition, the differences between the breeds were also related to solute transport, and lipid, carbohydrate and protein metabolism. The results provide key genes (such as *MYOD1, FOS*, *JAK2, MSTN,* and *DLK1*) and mechanisms (DE genes, pathways, and regulatory transcription factors) involved in the regulation of the progression of muscle development and intramuscular fat accretion, which also explain the phenotypic differences between the breeds. Taking into account the results of the two comparisons (age and breed effects), we can postulate that both breeds progress with different paucity regarding muscle development, proposing that myogenesis starts earlier, but progresses later and more slowly, in Iberian pigs. In other words, the Iberian breed is more precocious in muscle development than the Duroc breed at this specific growing stage. Our results highlight the deep influence of age and breed on muscle metabolism during growth, suggesting specific needs across the different phases of the growing period, which should be key in the design of breed- and age-specific productive feeding programs.

## Figures and Tables

**Table 1:** Calculated analysis ^1^, fatty acid composition and ingredients of the experimental diets.

Diets	Carbohydrate (CH) ^2^	High Oleic (HO) ^3^
Chemical composition, g/kg of feed		
Moisture	87.4	88.81
Lipids	24.53	77.65
Crude protein	156.00	156.00
Crude fiber	29.71	45.27
Nitrogen-free extractives	515.75	404.39
Ash	44.34	67.91
Main Fatty acids, g/kg of feed		
C14:0	0.14	0.13
C16:0	4.83	7.19
C18:0	0.84	1.83
C18:1 n-9	9.47	36.82
C18:2 n-6	14.24	16.68
C18:3 n-3	0.99	1.21
Ingredients, %		
Wheat	30	25
Wheat bran	--	7
Corn	33.90	--
Barley	16.87	38.87
Soybean meal (44%)	16.88	15.53
Calcium carbonate	0.83	0.75
Bicalcium phosphate	0.85	0.69
Marine Salt	0.40	0.40
Sepiolite	--	2.00
High oleic sunflower oil	--	6.00
L-Lysine	0.07	0.06
Vitamin and mineral premix	0.2	0.2

Both diets were isocaloric with 3.3 Kcal digestible energy; ^1^ according to Fundación Española Desarrollo Nutrición Animal (2010); ^2^ CH = carbohydrate diet without added fat; ^3^ HO = high oleic diet with high oleic sunflower oil.

**Table 2:** Meat quality data recorded in *biceps femoris* muscle of Iberian and Duroc pigs fed CH and HO diets, at an average LW of 51.2 kg (growing stage).

	Diet Effect			Breed Effect			Interaction Effect
Trait	CH (*n* = 22) Mean (s.e)	HO (*n* = 27) Mean (s.e)	*p*-Value	Iberian (*n* = 30) Mean (s.e)	Duroc (*n* = 19) Mean (s.e)	*p*-Value	*p*-Value Diet × Breed
Thawing Loss (%)	16.78(1.37)	16.26(1.32)	n.s.	16.18(1.10)	17.22(1.58)	n.s.	n.s.
Cooking Loss (%)	27.74(1.21)	28.65(1.17)	n.s.	25.63(0.98)	30.77(1.39)	0.005	n.s.
Shear Force (Kg/cm^2^)	4.35(0.26)	4.16(0.25)	0.07	3.87(0.25)	4.64(0.29)	0.05	0.03
L* Lightness	48.83(0.78)	48.38(0.75)	n.s.	49.29(0.67)	47.91(0.90)	0.09	n.s.
a* redness	5.53(0.39)	5.79(0.38)	n.s.	5.43(0.34)	5.88(0.46)	n.s.	n.s.
b* yellowness	6.27(0.16)	6.52(0.15)	n.s.	6.77(0.15)	6.02(0.18)	0.009	n.s.

**Table 3:** Most relevant canonical pathways activated in transition piglets and growers, related to cell growth and development.

**Pathways Activated in Transition Piglets**			
**Pathways**	**Related Functions**	***p*** **-Value**	**z-Score**
Oxidative Phosphorylation	Cell proliferation	0.001	−4.243
Cell cycle control of chromosomal replication	Cell proliferation	5.13 × 10^−7^	−2.668
PTEN Signaling	Cell proliferation and differentiation	0.003	−2.500
Estrogen-mediated S-phase Entry	Regulation of cell cycle	0.0001	−2.333
**Pathways Activated in Grower Pigs**			
**Pathways**	**Related Functions**	***p*** **-Value**	**z-Score**
NF-kB Signaling	Development and cell growth	0.005	4.379
Adrenomedullin signaling pathway	Angiogenesis	0.001	3.922
Melanocyte Development and Pigmentation Signaling	Development	6.3 × 10^−6^	3.578
NGF Signaling	Regulation of development and differentiation	1.2 × 10^−6^	3.411
ERK5 Signaling	Cell proliferation and differentiation	1.2 × 10^−6^	3.3
AMPK Signaling	Regulating growth and reprogramming metabolism	2.8 × 10^−5^	3.272
CNTF Signaling	Activates JAK-STAT signaling, related to myogenic differentiation	1.6 × 10^−5^	3.207
FGF Signaling	Regulation of proliferation, survival, migration and differentiation	0.004	3.207
p38 MAPK Signaling	Myogenic differentiation	0.008	3.207
Role of NANOG in Mammalian Embryonic Stem Cell Pluripotency	Embryonic development	0.0002	3.051
ErbB Signaling	Cell migration, proliferation, differentiation	0.002	2.84
Ephrin Receptor Signaling	Controls actin cytoskeleton dynamics	0.0005	2.837
HGF Signaling	Regulates cell growth, motility, and morphogenesis	0.0007	2.828
GM-CSF Signaling	Cell survival, proliferation and functional activation	0.006	2.714
IL-6 Signaling	Multifunctional cytokine that regulates organ development	0.0002	2.683
Sirtuin Signaling Pathway	Cell proliferation, differentiation, senescence, apoptosis, and metabolism	0.0008	2.611
IL-2 Signaling	Organ development	0.006	2.53
Thrombopoietin Signaling	Proliferation, differentiation and cell survival	0.008	2.53
EGF Signaling	Regulate cell growth, survival, proliferation and differentiation	0.0002	2.496
Antiproliferative Role of Somatostatin Receptor 2	Inhibition of proliferation and/or the induction of apoptosis via interactions with a GPRCs family	0.01	2.333
PDGF Signaling	Development, cell proliferation, migration and angiogenesis	0.0007	2.324
Prolactin Signaling	Growth and development	0.0001	2.138
Growth Hormone Signaling	Growth and development	0.0008	2.138
Signaling by Rho Family GTPases	Cytoskeleton signaling and ECM	0.0009	2.117
Apelin Endothelial Signaling Pathway	Angiogenesis	0.0004	2.065
Actin Cytoskeleton Signaling	Cytoskeleton signaling and ECM	0.005	2.041
JAK/Stat Signaling	Myogenic differentiation	9.1 × 10^−5^	2

**Table 4:** Most relevant activated upstream regulators (sorted by z-score) for the set of DEGs according to age (*p*-value < 0.05 and z-score > 3 or <−3).

Upstream Regulator	Molecule Type	Activation z-Score ^1^	*p*-Value	Molecules in Dataset	Related Functions
**Activated in Transition Piglets**					
CSF2	cytokine	−4.590	1.10 × 10^−8^	52	Inflammatory and immune response
ERBB2	kinase	−4.408	8.77 × 10^−9^	78	Cell growth and division
STK11	kinase	−4.207	3.20 × 10^−5^	32	Cell metabolism and energy homeostasis
MIR17HG	other	−3.756	4.70 × 10^−4^	21	Cell survival, proliferation, differentiation, and angiogenesis
EP400	other	−3.317	1.77 × 10^−10^	18	Cell proliferation
AREG	growth factor	−3.308	6.11 × 10^−6^	18	Regulation of cell population proliferation
PTGER2	G-protein-coupled receptor	−3.273	1.65 × 10^−5^	21	Regulation of cell population proliferation and inflammatory response
MITF	transcription regulator	−3.272	1.56 × 10^−6^	28	Regulation of melanocyte development
E2f	group	−3.259	5.45 × 10^−5^	20	Protein synthesis
RABL6	other	−3.176	4.00 × 10^−7^	18	Cell growth and survival
GMNN	transcription regulator	−3.162	0.01	12	Cell cycle control
**Activated in growers**					
RICTOR	other	4.730	0.03	30	Regulation of cell cycle
CD24	other	4.600	7.90 × 10^−10^	28	Modulation and differentiation of cell B growth
KLF4	transcription regulator	3.386	0.005	26	Down-regulation of cell proliferation
MEF2D	transcription regulator	3.238	0.0001	14	Control of muscle and neuronal cell differentiation
Irgm1	other	3.148	0.002	10	Innate immune response
TCF7L2	transcription regulator	3.072	3.05 × 10^−5^	46	Glucose metabolism
PDGF BB	complex	3.062	0.001	20	Regulate cell growth and division

^1^ Positive z-scores predict an overall increase in the activity of the regulator in growers, while negative z-scores indicate a prediction of an overall increase in the regulator activity in transition piglets.

**Table 5:** IPA-based list of pathways in the set of DEGs conditional on breed (*p*-value < 0.1, z-score > 2 or <−2).

Canonical Pathways	*p*-Value	Activation	z-Score	Molecules
GM-CSF Signaling	0.08	Iberian	−2.236	*CISH, JAK2, PIK3R1, RASD2, RUNX1, STAT1*
Growth Hormone Signaling	0.09	Iberian	−2.236	*FOS, GHR, IGF2, JAK2, PIK3R1, STAT1*
Retinol Biosynthesis	0.03	Duroc	2.236	*CEL, CES1, CES3, RBP7, RDH13*
LXR/RXR Activation	0.001	Duroc	2.111	*ARG2, FASN, IL18, IL1RAP, ITIH4, LDLR, LYZ, MLXIPL, NOR1, NOS2, PON3, RBP4, SERPINA1, TNFR1B*
Superpathway of Serine and Glycine Biosynthesis I	0.0001	Duroc	2.000	*PHGDH, PSAT1, PSPH, SHMT2*
Retinoate Biosynthesis I	0.05	Duroc	2.000	*DHRS7C, RBP7, RDH13, RDH16*

**Table 6:** IPA-based list of activated upstream regulators (sorted by z-score) for the set of DEGs according to breed (*p*-value < 0.01 and z-score > 2 or <−2).

Upstream Regulator	Molecule Type	Activation z-Score ^1^	*p*-Value	Molecules in Dataset	Related Functions
Activated in Iberian					
NUPR1	transcription regulator	−2.897	0.00005	10	Muscle cell apoptotic process
FOXO3	transcription regulator	−2.845	0.00006	10	Cellular response to glucose stimulus, hypoxia and oxidative stress. Control muscle growth
ALDH2	enzyme	−2.813	8.08 × 10^−7^	8	Carbohydrate metabolic, oxidation–reduction and apoptotic process
PDGF BB	complex	−2.764	0.0008	10	Proliferation and phosphorylation
HDL-cholesterol	complex	−2.400	0.006	6	Transport of lipids
GLI3	transcription regulator	−2.339	0.001	9	Developmental growth
F7	peptidase	−2.236	0.009	5	Regulation of leukocyte chemotaxis, protein processing, response to cholesterol and hypoxia
Rb	group	−2.219	0.0006	8	Cell differentiation and growth regulation of cell cycle
ICAM1	transmembrane receptor	−2.200	0.008	5	Cellular response to glucose stimulus, nutrient levels and hypoxia. Inflammatory response
MAP3K8	kinase	−2.145	0.002	10	MAPK and Insulin signaling, immune response and protein phosphorylation
IB3	kinase	−2.135	0.00001	8	Response to stress, insulin signaling, FA biosynthetic and lipid metabolic process
HDAC2	transcription regulator	−2.121	0.01	10	Circadian regulation of gene expression, muscle cell development and deacetylase activity
Activated in Duroc					
MITF	transcription regulator	2.688	0.0003	10	Development and growth
COL6A1	other	2.214	0.0003	5	Extracellular matrix organization and platelet-derived growth factor binding
IGF2BP1	translation regulator	2.200	0.009	5	Growth, regulator of IGF2 translation
COLQ	other	2.178	0.01	8	Extracellular matrix organization and protein binding
RABL6	other	2.121	0.009	8	Cell growth and GPT protein binding
UCP1	transporter	2.055	0.000008	10	Response to cold, fatty acid and ROS, long-chain fatty acid binding and oxidative phosphorylation
IDO1	enzyme	2.000	0.01	5	Inflammatory response, immune system process

^1^ Positive z-scores predict an overall increase in the activity of the regulator in Duroc pigs, while negative z-scores indicate a prediction of an overall increase in the regulator activity in Iberian pigs.

**Table 7:** Summary of differential expression results for the diet effect on transcriptome.

Group of Animals	No. DEGs	No. Upregulated in HO	Genes Upregulated in HO	FC Rank	No. Upregulated in CH	Genes Upregulated in CH	FC Rank
Iberian transition piglets	8	6	*FGG, FGB, ALB, FGF21, MIOX, CYP2D25*	5.3–38.3	2	*PHACTR3, INSIG1*	1.9–3.9
Iberian growers	8	4	*ENSSSCG00000003278, MYL4, ACP5, UNC93B1*	1.6–10.7	4	*PDE7B, DNAJB1, NEDD4L, ENSSSCG00000029160*	1.6–1.9
Duroc growers	3	1	*MYH11*	1.7	2	*SLA-5, MYL4*	3.5–4.4

**Table 8:** Technical validation of RNA-seq results by quantitative PCR (qPCR): genes, statistical significance and fold change values (FC) obtained with both techniques for the breed and age effects, and Pearson correlation between expression values obtained from both techniques.

**AGE EFFECT IN IBERIAN**	**RNA Seq (*n* = 12)**		**qPCR (*n* = 12)**			***p*-Value**
**Gene Symbol**	***p*-Value**	**Fold Change**	***p*-Value**	**Fold Change**	**Correlation (r)**	**(H0: r = 0)**
**Genes upregulated in piglets**						
*PVALB*	4.76 × 10^−7^	2.70	0.02	1.34	0.75	0.004
*DAPK3*	1.36 × 10^−6^	1.58	<0.0001	5.21	0.62	0.004
*GPX2*	2.80 × 10^−10^	7.24	0.01	1.93	0.86	5.81 × 10^−8^
*MTUS2*	4.94 × 10^−10^	3.57	0.01	1.82	0.61	0.002
*MGLL*	0.05	1.24	0.4	1.34	0.58	0.03
**Genes upregulated in growers**						
*ME1*	3.10 × 10^−4^	0.59	0.02	0.59	0.84	2.15 × 10^−7^
*LEP*	0.001	0.33	0.0009	0.32	0.82	1.11 × 10^−6^
*MSTN*	5.00 × 10^−5^	0.44	0.01	0.7	0.83	4.08 × 10^−7^
*IGF2*	0.43	0.88	0.7	0.96	0.52	0.01
*FASN*	0.01	0.48	0.06	0.73	0.78	8.04 × 10^−6^
*NR4A3*	0.59	0.76	0.5	0.67	0.94	1.84 × 10^−11^
*PON3*	0.009	0.75	0.002	0.63	0.7	0.01
**BREED EFFECT**	**RNA Seq (*n* = 12)**		**qPCR (*n* = 12)**			***p*-Value**
**Gene Symbol**	***p*-Value**	**Fold Change**	***p*-Value**	**Fold Change**	**Correlation (r)**	**(H0: r = 0)**
**Genes upregulated in Iberian pigs**						
*FASN*	0.01	1.99	0.08	1.21	0.77	1.03 × 10^−5^
*ME1*	4.45 × 10^−5^	1.62	0.02	1.57	0.84	2.44 × 10^−7^
*NR4A3*	0.01	2.74	0.05	1.48	0.90	1.76 × 10^−9^
*PON3*	4.59 × 10^−10^	12.34	<0.0001	5.30	0.89	4.77 × 10^−9^
*LEP*	0.07	1.62	0.08	1.15	0.81	1.71 × 10^−6^
*MSTN*	0.06	1.37	0.2	1.08	0.78	6.31 × 10^−6^
**Genes upregulated in Duroc pigs**						
*IGF2*	1.92 × 10^−7^	0.33	<0.0001	0.40	0.83	5.06 × 10^−7^
*PVALB*	2.62 × 10^−6^	0.42	0.0008	0.55	0.68	0.0003
*DAPK3*	1.61 × 10^−13^	0.53	0.001	0.58	0.73	4.69 × 10^−5^
*GPX2*	4.78 × 10^−10^	0.12	0.003	0.49	0.80	2.50 × 10^−6^
*MTUS2*	2.52 × 10^−18^	0.31	<0.0001	0.30	0.78	6.16 × 10^−6^
*MGLL*	0.0001	0.66	0.03	0.73	0.69	0.0003

## Data Availability

The results from data analyses performed in this study are included in this article and its tables. Raw sequencing data are available from the corresponding author on reasonable request.

## Supplementary Materials

The following are available online at https://www.mdpi.com/article/10.3390/ani11123505/s1: Table S1: diet composition; Table S2: primer design for qPCR validation; Table S3: fatty acid composition; Table S4: DEGs age effect; Table S5: biological functions age effect; Table S6: canonical pathways age effect; Table S7: upstream regulators age effect; Table S8: DEGs breed effect; Table S9: biological functions breed effect; Table S10: canonical pathways breed effect; Table S11: upstream regulators breed effect; Table S12: common genes between DEGs affected by age and breed effects; Table S13: DEGs diet effect; Table S14: DESeq2 results for the different interaction effects.
