# Supplementary material for: Changes in Biceps femoris Transcriptome along Growth in Iberian Pigs Fed Different Energy Sources and Comparative Analysis with Duroc Breed
Source: Animals (Basel). 2021 Dec 8;11(12):3505. doi: 10.3390/ani11123505 (PMC8697974; doi:10.3390/ani11123505)
Supplement: Supplementary file 1 [file animals-11-03505-s001.zip › Table S1_Primers design for qPCR validation.pdf]

Table S1: Primer design for qPCR and PCR efficiencies

Gene symbol	Gene name	GenBank ID	Forward primer sequence	Reverse primer sequence	Efficency (%)
GPX2	Glutathione peroxidase 2	NM_001115136	CTATCAGCCTGGACGGGGAGAA	AGGGCGGACGTACTTGAGGCTGTT	86
ME1	Malic enzyme 1	XM_001924333.4	GCCGGCTTTATCCTCCTCT	TCAAGTTTGGTCTGTATTTTCTGG	87
DAPK3	Death associated protein kinase 3	XM_021084109	ACTTCATCCGCCGGCTGCTTGTCA	GGCTTCCGGCTGCTGTCCTCACGA	81
MTUS2	Microtubule associated scaffold protein 2	XM_021065171	TCATTACAGATCGCGCTGGC	GGTGTTCTGCTCATCCTGG	82
MSTN	Myostatin	NM_214435.2	CCACTCCGGGAACTGATTGAT	GTTGGGCTTTACTACTTTATTGT	75
NR4A3	Nuclear receptor subfamily 4 group A member 3	NM_214247.1	AAAAACTGCCCCGTAGACAAGAGG	TCGGACAAGGGCATTCATCATACA	86
LEP	Leptin	NM_213840.1	GGCCCTATCTGTCCTACGTTGAAG	TGGAAGGCAGACTGGTGAGGAT	77
FASN	Fatty acid synthase	NM_001099930.1	GCAGGCGCGTGATGGGAATGGTG	GCCCGAGCCCGAGTGGATGAGCA	77
PON3	Paraoxonase 3	NP_001038069.1	ACGGGAGATATTTGGGCAGG	TGTTGGCATACTCGGTGGTT	79
MGLL	Monoglyceride lipase	NM_001143718.1	CCCCGGCGGACCCACAGA	GGTAGGGCTGGGGCTGCTGGTGTT	78
IGF2	Insulin like growth factor 2	NM_001190157.1	GCCGCTGCTCGTGCTGCTCTT	GCTTGGCCGGGCCTGCTGAA	78
PVALB	Parvalbumin	NM_213883.2	AAGACGCTGATGGCTGCTGGAGAC	ATAAGGGATGGGGGAGTAAAA	78
ACTB	Beta-actin	XM_003124280.4	TCTGGCACCACACCTTCT	TGATCTGGGTCATCTTCTCAC	90
PPIA	Peptidylprolyl isomerase A	NM_214353	GGGAGAAAGGATTTGGTTAT	ATGGACAAGATGCCAGGAC	95
